# Atrial fibrillation increases left and right atrial pressures in patients with chronic heart diseases

**DOI:** 10.1093/eschf/xvaf032

**Published:** 2026-01-08

**Authors:** Davide Genovese, Michele Strosio, Enrico Fantini, Giacomo Prete, Marco Previtero, Carlo Cernetti, Giuseppe Tarantini, Domenico Corrado, Martina Perazzolo Marra

**Affiliations:** Cardiology Unit, Department of Cardiac-Thoracic-Vascular Sciences and Public Health, University of Padova, Via Giustiniani, 2, Padova 35128, Italy; Cardiology Unit, Cardio-Neuro-Vascular Department, Ca’ Foncello Hospital, Piazzale Ospedale, 1, Treviso 31100, Italy; Cardiology Unit, Department of Cardiac-Thoracic-Vascular Sciences and Public Health, University of Padova, Via Giustiniani, 2, Padova 35128, Italy; Cardiology Unit, Department of Cardiac-Thoracic-Vascular Sciences and Public Health, University of Padova, Via Giustiniani, 2, Padova 35128, Italy; Cardiology Unit, Cardio-Neuro-Vascular Department, Ca’ Foncello Hospital, Piazzale Ospedale, 1, Treviso 31100, Italy; Cardiology Unit, Cardio-Neuro-Vascular Department, Ca’ Foncello Hospital, Piazzale Ospedale, 1, Treviso 31100, Italy; Cardiology Unit, Department of Cardiac-Thoracic-Vascular Sciences and Public Health, University of Padova, Via Giustiniani, 2, Padova 35128, Italy; Cardiology Unit, Cardio-Neuro-Vascular Department, Ca’ Foncello Hospital, Piazzale Ospedale, 1, Treviso 31100, Italy; Cardiology Unit, Department of Cardiac-Thoracic-Vascular Sciences and Public Health, University of Padova, Via Giustiniani, 2, Padova 35128, Italy; Cardiology Unit, Department of Cardiac-Thoracic-Vascular Sciences and Public Health, University of Padova, Via Giustiniani, 2, Padova 35128, Italy; Cardiology Unit, Department of Cardiac-Thoracic-Vascular Sciences and Public Health, University of Padova, Via Giustiniani, 2, Padova 35128, Italy

**Keywords:** Right atrial pressure, Left atrial pressure, Pulmonary capillary wedge pressure, Right heart catheterization, Atrial fibrillation, Heart failure

## Abstract

**Introduction:**

Pulmonary capillary wedge pressure (PCWP) and right atrial pressure (RAP) are key haemodynamic indicators of cardiac congestion. Atrial fibrillation (AF) often coexists with several heart diseases, making it challenging to determine AF’s independent contribution to atrial pressure elevation. Therefore, the impact of AF on PCWP and RAP requires clarification. We sought to quantify the contribution of AF on PCWP and RAP within patients with various chronic heart diseases.

**Methods:**

We performed a single-center retrospective analysis on 1452 patients (age: 68.0 ± 13.6 years, 58.7% male, 26% AF) with chronic heart diseases undergoing right heart catheterization (RHC). PCWP and RAP were measured during RHC, and the underlying AF or sinus rhythm (SR) was annotated. To isolate AF effect from clinical, haemodynamic, and echocardiographic confounders, two propensity score matching analyses yielded two balanced cohorts for PCWP (*n* = 496) and RAP (*n* = 494) analysis.

**Results:**

After matching, PCWP was higher in the AF than the SR group (18.4 ± 0.49 mmHg vs 15.7 ± 0.49 mmHg; *P* < .001). Similarly, RAP was higher in the AF than the SR group (8.7 ± 0.34 mmHg vs 7.5 ± 0.34 mmHg; *P* = .02). The findings were highly robust for PCWP (*E*-value = 10.8) and moderately robust for RAP (*E*-value = 2.78) to unmeasured confounders. Additionally, patients in the SR cohort with a prior history of AF had significantly higher PCWP and RAP compared to patients with no AF history.

**Conclusion:**

In our cohort, AF increased PCWP by 2.6 mmHg and RAP by 1.1 mmHg. Furthermore, a previous history of AF is linked to higher atrial pressures in patients later in SR.

## Graphical Abstract

**Graphical Abstract:**
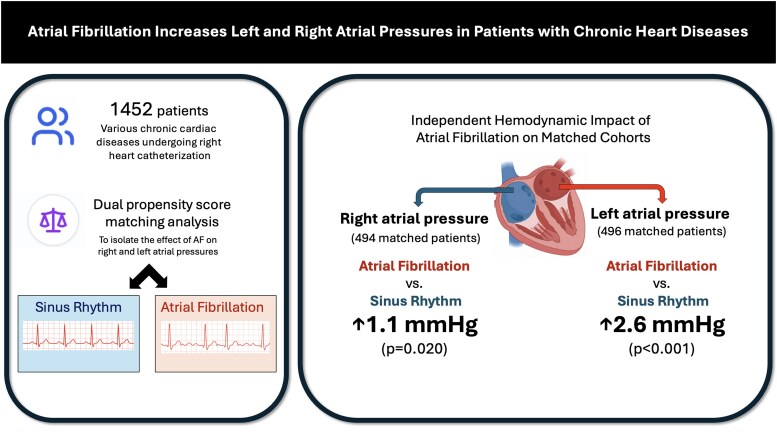

## Background

Pulmonary capillary wedge pressure (PCWP) and the right atrial pressure (RAP) are fundamental haemodynamic parameters for cardiac patients.^1^ PCWP integrates the haemodynamic effects of LV diastolic function, mitral valve function, and LA function on the pulmonary vascular system, whether RAP reflects the hzemodynamic burden of RV diastolic function, tricuspid valve function, and RA function on the central venous system.^2^ Indeed, they provide critical insights into volume status and the degree of pulmonary and systemic venous congestion, carrying substantial diagnostic and prognostic information.^3,4^ Notably, an increase in either PCWP or RAP is the haemodynamic hallmark of left and right heart failure (HF).^5^

Atrial fibrillation (AF) represents a major public health burden due to its strong association with stroke, HF hospitalizations, and increased mortality.^6–8^ AF can worsen underlying cardiac conditions through several mechanisms: loss of atrial contribution to ventricular filling, rapid and irregular ventricular rates impairing diastolic filling time and systolic performance, and potential development of tachycardia-induced cardiomyopathy.^9^ While AF is associated with elevated atrial pressures, it often develops in the context of underlying structural heart diseases, which independently promote atrial remodelling and increase filling pressures.^10–13^ This overlap complicates assessing atrial pressure increase solely due to AF. Distinguishing AF's independent effect on atrial pressures from other cardiac conditions is crucial for understanding its pathophysiology and interpreting haemodynamic data accurately. Therefore, the objective of this study was to quantify, through a robust, dual-model propensity score matching analysis, the independent effect of AF on PCWP and RAP within a large group of patients with various chronic heart conditions and to examine the relationship between a prior history of AF and the extent of changes in atrial pressures.

## Methods

### Study design and population

This single-center, retrospective, observational study was conducted at the Department of Cardiac, Thoracic, Vascular Sciences and Public Health, University Hospital of Padua, Italy. The study cohort was derived from an institutional registry of consecutive adult patients who underwent clinically indicated right heart catheterization (RHC) to evaluate chronic heart conditions between May 2015 and March 2022. From an initial cohort of 1562 patients identified from the registry, individuals were excluded if they had congenital heart disease (*n* = 55), prosthetic mitral valves (*n* = 40), or prosthetic tricuspid valves (*n* = 15), owing to the distinct pathophysiology and potential confounding effects of these conditions on atrial pressures. The final study population comprised 1452 patients. The study protocol was approved by the local Ethics Committee (Project N. 521n/AO/24; Protocol N. 0066025) and conducted following the Declaration of Helsinki. All enrolled patients provided written informed consent to use their anonymized clinical data for observational research. The datasets generated and/or analysed during the current study are available from the corresponding author upon reasonable request.

### Data collection

Relevant clinical, laboratory, haemodynamic, and echocardiographic data were retrospectively collected from institutional electronic medical records, dedicated catheterization laboratory databases, and echocardiography reporting systems.

#### Clinical and laboratory data

Baseline demographic and clinical information, history of AF (paroxysmal, persistent, or permanent), and cardiovascular medications were collected. Key laboratory parameters were obtained from venous blood samples drawn within 24 h of the RHC procedure.

#### Haemodynamic data and cardiac rhythm classification

Right heart catheterization was performed using a standard Swan-Ganz catheter (SGC) introduced via a femoral venous approach, following conventional methodology. The external fluid-filled pressure transducer was zeroed at the mid-thoracic line with the patient in the supine position. The catheter was advanced under fluoroscopic guidance. For PCWP measurement, the SGC balloon was inflated and advanced until reaching the pulmonary wedge position; correct placement was confirmed by both fluoroscopy and characteristic pressure waveform morphology. PCWP measurements were recorded at end-expiration, averaging mean values over a minimum of five consecutive cardiac cycles. Subsequently, the balloon was deflated, and the catheter tip was progressively retracted to the pulmonary artery, right ventricle, and finally into the right atrium. The right atrial position was identified by fluoroscopy and pressure waveform. RAP measurements were obtained from pressure-time recordings at the end of normal expiration, averaging mean values over at least five cardiac cycles. Other invasively measured parameters collected were right ventricular end-diastolic pressure (RVEDP), pulmonary artery systolic pressure (PASP), left ventricular end-diastolic pressure (LVEDP, obtained during simultaneous left heart catheterization in all patients), and cardiac index (CI, calculated using the indirect Fick method).

The cardiac rhythm (AF or SR) present during the RHC procedure was assessed from continuous electrocardiographic monitoring recorded simultaneously with the haemodynamic measurements and collected for analysis from the medical records.

#### Echocardiographic data

Comprehensive transthoracic echocardiograms (TTE) were performed according to ASE/EACVI recommendations^14,15^ and analysed offline using dedicated software (ComPACS; MediMatic Srl). Key measurements retrospectively collected for analysis included left ventricular ejection fraction (LVEF, calculated using the biplane Simpson's method), maximum left atrial volume index (LAVi), maximum right atrial volume (RAVi), right ventricular fractional area change (RVFAC), and mitral and tricuspid regurgitation (MR and TR) severity and mitral valve stenosis (MS) severity.

### Statistical analysis

Continuous variables were expressed as mean ± standard deviation (SD) or median [interquartile range (IQR)] as appropriate. Categorical variables were presented as counts and percentages. Missing echocardiographic and laboratory data (<5%) were handled with multiple imputation. Propensity score (PS) matching^16^ was performed to address potential confounding and isolate the independent effect of AF on atrial pressures. Two separate PSs were generated using multivariable logistic regression models: one predicting the likelihood of AF for the PCWP analysis and another for the RAP analysis, accounting for the potential different confounders shared by AF with PCWP and RAP, respectively. We chose this dual-model approach because several confounders for PCWP are distinct from those for RAP. This approach ensured optimal, specific covariate balance for each atrial pressure, rather than relying on a single model subject to suboptimal balance and residual confounding. Therefore, for each one of the PCWP and RAP analyses, a PS matching analysis was applied, and patients with AF were matched 1:1 to patients in SR using a nearest-neighbor algorithm, without replacement, with a calipre width of 0.2 standard deviations of the logit of the PSs. Standardized mean differences (SMD) were calculated for all covariates included in the PS models before and after matching to assess balance, with an SMD <0.10 indicating adequate balance. In the matched cohorts, differences in PCWP and RAP between the AF and SR groups were assessed using linear regression models. The estimated marginal means (EMMs) for atrial pressures were calculated and compared between the matched AF and SR groups to determine the differences for PCWP and RAP due to AF. Despite PWCP and RAP being right-skewed, we did not perform a log transformation due to the large sample size that ensures the robustness of the estimates. E-values were calculated to assess the robustness of the findings to potential unmeasured confounding.^17^ We conducted a subgroup analysis to test the influence of confounders on the impact of AF on atrial pressures. Furthermore, separate multiple linear regression analyses were performed for the AF and SR subgroups to explore the association between the history of AF and PCWP and RAP values, adjusting for relevant covariates.

All statistical analyses were performed using R version 4.4.2 (R Foundation for Statistical Computing, Vienna, Austria). A two-sided *P*-value <.05 was considered statistically significant.

## Results

### Baseline characteristics of the overall cohort

The final study population comprised 1452 patients. The mean age was 68.0 ± 13.6 years, and 58.7% were male. The population represented a broad spectrum of chronic heart conditions, with the most common primary indications for RHC being aortic stenosis (33.6%), dilated cardiomyopathy (15.6%), ischaemic heart disease (13.4%), and significant mitral regurgitation (10.9%). Baseline clinical, laboratory, haemodynamic, and echocardiographic characteristics are presented in *Table 1*.

**Table 1:** Baseline characteristics of the study population and comparison between atrial fibrillation and SR groups

Characteristic	Overall *N* = 1452	Sinus rhythm *N* = 1074	Atrial fibrillation *N* = 378	*P*-value
**Age (years)**	68.0 ± 13.6	66.6 ± 14.2	72.0 ± 11.1	**<.001**
**Gender (male)**	852 (58.7%)	616 (57.4%)	236 (62.4%)	.085
**BMI (kg/m²)**	26.4 ± 4.8	26.3 ± 4.8	26.8 ± 4.7	**.009**
**Cardiac^a^**				
IHD	194 (13.4%)	147 (13.7%)	47 (12.4%)	**<**.**001**
DCM	227 (15.6%)	156 (14.5%)	71 (18.8%)	
PH	139 (9.6%)	126 (11.7%)	13 (3.4%)	
AS	488 (33.6%)	387 (36.0%)	101 (26.7%)	
AR	61 (4.2%)	57 (5.3%)	4 (1.1%)	
MS	60 (4.1%)	24 (2.2%)	36 (9.5%)	
MR	158 (10.9%)	109 (10.1%)	49 (13.0%)	
TR	45 (3.1%)	14 (1.3%)	31 (8.2%)	
HCM	33 (2.3%)	21 (2.0%)	12 (3.2%)	
ARVC	14 (1.0%)	11 (1.0%)	3 (0.8%)	
Others	33 (2.3%)	22 (2.0%)	11 (2.9%)	
**PM**	144 (9.9%)	75 (7.0%)	69 (18.3%)	**<.001**
**AF Type**				
None	877 (60.4%)	877 (81.7%)	0 (0.0%)	**<.001**
Paroxysmal	230 (15.8%)	170 (15.8%)	60 (15.9%)	
Persistent	66 (4.5%)	27 (2.5%)	39 (10.3%)	
Permanent	279 (19.2%)	0 (0.0%)	279 (73.8%)	
**Haemoglobin (g/dL)**	12.9 ± 3.9	13.0 ± 4.4	12.8 ± 1.9	.772
**Creatinine (µmol/L)**	98.3 ± 69.4	96.9 ± 76.7	102.4 ± 42.0	**<.001**
**Albumin (g/L)**	37.9 ± 4.8	38.0 ± 4.8	37.5 ± 4.9	**.039**
**Beta-blocker**	864 (59.5%)	588 (54.7%)	276 (73.0%)	**<.001**
**ACEi/ARB/ARNI**	847 (58.3%)	606 (56.4%)	241 (63.8%)	**.013**
**Furosemide (mg/die)**	48.4 ± 98.3	37.1 ± 82.2	80.3 ± 128.7	**<.001**
**SBP (mmHg)**	130.5 ± 26.7	132.0 ± 27.5	126.3 ± 24.1	**<.001**
**HR (bpm)**	75.8 ± 14.7	74.5 ± 14.1	79.3 ± 15.8	**<.001**
**RAP (mmHg)**	6.8 ± 4.6	6.0 ± 4.1	9.0 ± 5.1	**<.001**
**RVEDP (mmHg)**	8.4 ± 5.3	8.0 ± 5.4	9.6 ± 5.0	**<.001**
**PASP (mmHg)**	37.5 ± 16.0	36.1 ± 16.1	41.6 ± 15.0	**<.001**
**PCWP (mmHg)**	14.5 ± 7.7	13.0 ± 7.4	18.7 ± 7.0	**<.001**
**CI (L/min/m²)**	2.8 ± 0.7	2.9 ± 0.8	2.5 ± 0.6	**<.001**
**LVEDP (mmHg)**	17.5 ± 7.5	17.6 ± 7.6	17.0 ± 7.0	.176
**LVEF (%)**	49.8 ± 15.4	51.1 ± 15.1	46.1 ± 15.6	**<.001**
**LAVi (mL/m²)**	52.0 ± 27.6	45.0 ± 18.0	71.9 ± 38.4	**<.001**
**RAVi (mL/m²)**	39.3 ± 24.7	32.5 ± 16.4	58.8 ± 32.8	**<.001**
**RVFAC (%)**	39.1 ± 10.1	40.4 ± 9.7	35.4 ± 10.1	**<.001**
**Mitral regurgitation**				
None/trivial	476 (32.8%)	397 (37.0%)	79 (20.9%)	**<**.**001**
Mild	506 (34.8%)	375 (34.9%)	131 (34.7%)	
Mild/moderate	107 (7.4%)	67 (6.2%)	40 (10.6%)	
Moderate	144 (9.9%)	82 (7.6%)	62 (16.4%)	
Moderate/severe	64 (4.4%)	41 (3.8%)	23 (6.1%)	
Severe	155 (10.7%)	112 (10.4%)	43 (11.4%)	
**Tricuspid regurgitation**				
None/trivial	485 (33.4%)	435 (40.5%)	50 (13.2%)	**<**.**001**
Mild	553 (38.1%)	427 (39.8%)	126 (33.3%)	
Mild/moderate	117 (8.1%)	66 (6.1%)	51 (13.5%)	
Moderate	141 (9.7%)	78 (7.3%)	63 (16.7%)	
Moderate/severe	43 (3.0%)	25 (2.3%)	18 (4.8%)	
Severe	113 (7.8%)	43 (4.0%)	70 (18.5%)	
**Mitral stenosis**				
None	1198 (82.5%)	914 (85.1%)	284 (75.1%)	**<**.**001**
Mild	149 (10.3%)	103 (9.6%)	46 (12.2%)	
Moderate	63 (4.3%)	38 (3.5%)	25 (6.6%)	
Severe	42 (2.9%)	19 (1.8%)	23 (6.1%)	

ACEi, ACE inhibitors; AF, atrial fibrillation; ARB, angiotensin receptor blocker; ARNI, angiotensin receptor neprilysin inhibitor; ARVC, arrhythmogenic right ventricular cardiomyopathy; AS, aortic stenosis; AR, aortic regurgitation; BMI, body mass index; CI, Cardiac Index; DCM, dilated cardiomyopathy; HCM, hypertrophic cardiomyopathy; HR, heart rate; IHD, ischaemic heart disease; LAVi, left atrial maximal volume; LVEDP, left ventricular end-diastolic pressure; LVEF, left ventricular ejection fraction; MR, mitral regurgitation; MS, mitral stenosis; SBP, systolic blood pressure; PASP, pulmonary artery systolic pressure; PCWP, pulmonary capillary wedge pressure; PH, pulmonary hypertension; PM, pacemaker; RAP, right atrial pressure; RVEDP, right ventricular end-diastolic pressure; RVFAC, right ventricular fractional area change; RAVi right atrial maximal volume; TR, tricuspid regurgitation.

Values reported as mean ± SD or *n* (%).

Bold denoted statistically significant (*P* < 0.05).

^a^In patients with multiple cardiac diseases, only the primary condition leading to an RHC exam was reported for each patient.

### Comparison of baseline characteristics before matching

Patients were stratified according to the cardiac rhythm documented during RHC: 1074 (74.0%) patients were in SR, and 378 (26.0%) were in AF. Before PS matching, numerous significant differences were observed between the two groups (*Table 1*). Patients with AF were significantly older (72.0 ± 11.1 vs 66.6 ± 14.2 years, *P* < .001), had higher BMI (26.8 ± 4.7 vs 26.3 ± 4.8 kg/m², *P* = .009), higher prevalence of previous pacemaker implantation (18.3% vs 7.0%, *P* < .001), received higher doses of furosemide (mean: 80.3 vs 37.1 mg/day, *P* < .001), were more likely to be on betablockers (73.0% vs 54.7%, *P* < .001) and ACEi/ARB/ARNI (63.8% vs 56.4%, *P* = .013). Echocardiographically, patients with AF had lower LVEF (46.1 ± 15.6% vs 51.1 ± 15.1%, *P* < .001), larger LAVi (71.9 ± 38.4 vs 45.0 ± 18.0 mL/m², *P* < .001), larger RAVi (58.8 ± 32.8 vs 32.5 ± 16.4 mL/m², *P* < .001), and worse RVFAC (35.4 ± 10.1% vs 40.4 ± 9.7%, *P* < .001). Haemodynamically, patients in AF had faster HR (79.3 ± 15.8 vs 74.5 ± 14.1 bpm, *P* < .001), lower CI (2.5 ± 0.6 vs 2.9 ± 0.8 L/min/m², *P* < .001), and higher PCWP (18.7 ± 7.0 mmHg vs 13.0 ± 7.4 mmHg, *P* < .001) and RAP (9.0 ± 5.1 mmHg vs 6.0 ± 4.1 mmHg, *P* < .001) compared to the SR group.

### Propensity score matching and matched cohort characteristics

Two separate PS matching analyses were performed to create two dedicated cohorts to control for the confounders shared by AF and, respectively, PCWP and RAP.

#### Pulmonary capillary wedge pressure–matched cohort

Propensity score Matching yielded 248 pairs (*N* = 248 in SR group, *N* = 248 in AF group). Baseline characteristics for this matched cohort are displayed in *Table 2*. The matching procedure achieved an excellent balance with SMDs < 0.1 for all confounders included in the PS model (age, BMI, cardiac diagnosis, PM, haemoglobin, creatinine, albumin, beta-blocker, ACEi/ARB/ARNI, furosemide, SBP, HR, CI, LVEDP, LVEF, LAVi, MR, and MS severity), indicating negligible residual differences in baseline characteristics between the matched SR and AF groups (Supplementary data online, *Figs. S1* and *S2*).

**Table 2:** Assessment of covariate balance before and after PS matching for pulmonary capillary wedge pressure analysis

	Before matching			After matching		
	Atrial fibrillation *n* = 378	Sinus rhythm *n* = 1074	Std. mean diff.	Atrial fibrillation *n* = 248	Sinus rhythm *n* = 248	Std. mean diff.
**Distance**	0.5471	0.1594	13.790	0.4224	0.3999	0.0802
**Age (years)**	72.0	66.6	0.4865	72.1	71.8	0.0291
**BMI (kg/m²)**	26.8	26.3	0.1168	27.0	27.0	−0.0141
**Cardiac diagnosis**						
IHD	12.4	13.7	−0.0380	14.5	13.3	0.0367
DCM	18.8	14.5	0.1090	18.6	21.4	−0.0723
PH	3.4	11.7	−0.4551	4.0	3.2	0.0443
AS	26.7	36.0	−0.2105	30.7	34.3	−0.0820
AR	1.1	5.3	−0.4153	1.6	1.6	0.0000
MS	9.5	2.2	0.2483	7.3	5.2	0.0687
MR	13.0	10.2	0.0838	12.5	13.3	−0.0240
TR	8.2	1.3	0.2514	4.8	3.2	0.0588
HCM	3.2	2.0	0.0695	3.2	1.6	0.0920
ARVC	0.8	1.0	−0.0260	1.2	0.8	0.0454
Others	2.9	2.1	0.0513	1.6	2.0	−0.0240
**PM**						
No	81.8	93.0	−0.2918	84.7	83.5	0.0313
Yes	18.3	7.0	0.2918	15.3	16.5	−0.0313
**Haemoglobin (g/dL)**	12.8	13.0	−0.0710	12.9	13.0	−0.0660
**Creatinine (µmol/L)**	102.4	96.9	0.1314	99.0	101.7	−0.0640
**Albumin (g/L)**	37.5	38.0	−0.1046	37.5	37.2	0.0580
**Beta-blocker**						
No	27.0	45.3	−0.4115	29.8	30.7	−0.0182
Yes	73.0	54.8	0.4115	70.2	69.4	0.0182
**ACEi/ARB/ARNI**						
No	36.2	43.6	−0.1525	35.1	34.7	0.0084
Yes	63.8	56.4	0.1525	64.9	65.3	−0.0084
**Furosemide (mg/die)**	80.3	37.1	0.3353	73.9	73.9	0.0006
**SBP (mmHg)**	126.3	132.0	−0.2355	129.8	131.0	−0.0493
**HR (bpm)**	79.3	74.5	0.3052	78.2	77.4	0.0506
**CI (L/min/m²)**	2.5	2.9	−0.5453	2.5	2.5	−0.0178
**LVEDP (mmHg)**	17.0	17.6	−0.0928	17.7	17.8	−0.0087
**LVEF (%)**	46.1	51.1	−0.3164	46.9	46.7	0.0174
**LAVi (mL/m²)**	71.9	45.0	0.6996	62.7	60.6	0.0555
**Mitral regurgitation**						
None/trivial	20.9	37.0	−0.3951	22.6	23.0	−0.0099
Mild	34.7	34.9	−0.0055	34.7	33.1	0.0339
Mild/moderate	10.6	6.2	0.1412	10.5	10.5	0.0000
Moderate	16.4	7.6	0.2368	14.1	14.9	−0.0218
Moderate/severe	6.1	3.8	0.0948	6.9	5.7	0.0506
Severe	11.4	10.4	0.0298	11.3	12.9	−0.0508
**Mitral stenosis**						
None	75.1	85.1	−0.2307	75.4	76.2	−0.0187
Mild	12.2	9.6	0.0789	12.9	13.3	−0.0123
Moderate	6.6	3.5	0.1238	6.5	6.1	0.0162
Severe	6.1	1.8	0.1805	5.2	4.4	0.0337

ACEi, ACE inhibitors; AF, atrial fibrillation; ARB, angiotensin receptor blocker; ARNI, angiotensin receptor neprilysin inhibitor; ARVC, arrhythmogenic right ventricular cardiomyopathy; AS, aortic stenosis; AR, aortic regurgitation; BMI, body mass index; CI, Cardiac Index; DCM, dilated cardiomyopathy; HCM, hypertrophic cardiomyopathy; HR, heart rate; IHD, ischaemic heart disease; LAVi, left atrial maximal volume; LVEDP, left ventricular end-diastolic pressure; LVEF, left ventricular ejection fraction; MR, mitral regurgitation; MS, mitral stenosis; SBP, systolic blood pressure; PASP, pulmonary artery systolic pressure; PCWP, pulmonary capillary wedge pressure; PH, pulmonary hypertension; PM, pacemaker; RAP, right atrial pressure; RVEDP, right ventricular end-diastolic pressure; RVFAC, right ventricular fractional area change; RAVi right atrial maximal volume; TR, tricuspid regurgitation.

#### Right atrial pressure–matched cohort

A separate PS matching analysis for RAP yielded 247 pairs (*N* = 247 in SR group, *N* = 247 in AF group). Baseline characteristics for this cohort are shown in *Table 3*. Similar to the PCWP cohort, excellent covariate balance was achieved with SMDs < 0.1 for all confounders included in the PS model (ae, BMI, cardiac diagnosis, PM, haemoglobin, creatinine, albumin, beta-blockers, ACEi/ARB/ARNI, furosemide, PASP, HR, CI, RVEDP, RVFAC, RAVi, TR severity) (Supplementary data online, *Figs. S3* and *S4*).

**Table 3:** Assessment of covariate balance before and after PS matching for right atrial pressure analysis

	Before matching			After matching		
	Atrial fibrillation *n* = 378	Sinus rhythm *n* = 1074	Std. mean diff.	Atrial fibrillation *n* = 247	Sinus rhythm *n* = 247	Std. mean diff.
**Distance**	0.5637	0.1535	14.807	0.4378	0.4102	0.0999
**Age (years)**	72.0	66.6	0.4865	71.1	70.1	0.0901
**BMI (kg/m²)**	26.8	26.3	0.1168	27.3	27.3	0.0032
**Cardiac diagnosis**						
IHD	12.4	13.7	−0.0380	15.4	13.0	0.0736
DCM	18.8	14.5	0.1090	22.3	23.9	−0.0415
PH	3.4	11.7	−0.4551	3.6	4.9	−0.0666
AS	26.7	36.0	−0.2105	29.6	28.3	0.0274
AR	1.1	5.3	−0.4153	1.6	2.0	−0.0396
MS	9.5	2.2	0.2483	4.5	6.1	−0.0552
MR	13.0	10.2	0.0838	12.6	11.7	0.0241
TR	8.2	1.3	0.2514	4.9	4.1	0.0295
HCM	3.2	2.0	0.0695	2.0	2.0	0.0000
ARVC	0.8	1.0	−0.0260	0.4	0.8	−0.0456
Others	2.9	2.1	0.0513	3.2	3.2	0.0000
**PM**						
No	81.8	93.0	−0.2918	83.0	85.0	−0.0524
Yes	18.3	7.0	0.2918	17.0	15.0	0.0524
**Haemoglobin (g/dL)**	12.8	13.0	−0.0710	12.9	12.7	0.0766
**Creatinine (µmol/L)**	102.4	96.9	0.1314	103.2	102.4	0.0201
**Albumin (g/L)**	37.5	38.0	−0.1046	37.5	37.3	0.0433
**Beta-blocker**						
No	27.0	45.3	−0.4115	29.2	29.6	−0.0091
Yes	73.0	54.8	0.4115	70.9	70.5	0.0091
**ACEi/ARB/ARNI**						
No	36.2	43.6	−0.1525	37.3	35.6	0.0337
Yes	63.8	56.4	0.1525	62.8	64.4	−0.0337
**Furosemide (mg/die)**	80.3	37.1	0.3353	78.9	73.5	0.0420
**HR (bpm)**	79.3	74.5	0.3052	79.6	79.1	0.0321
**RVEDP (mmHg)**	9.6	8.0	0.3279	9.3	9.6	−0.0587
**PASP (mmHg)**	41.6	36.1	0.3617	40.7	41.9	−0.0792
**CI (L/min/m²)**	2.5	2.9	−0.5453	2.6	2.6	0.0115
**RAVi (mL/m²)**	58.8	32.5	0.8021	50.2	47.3	0.0882
**RVFAC (%)**	35.4	40.4	−0.5044	35.9	35.8	0.0191
**Tricuspid regurgitation**						
None/trivial	13.2	40.5	−0.8051	17.8	15.0	0.0837
Mild	33.3	39.8	−0.1363	38.5	39.3	−0.0172
Mild/moderate	13.5	6.2	0.2150	11.7	12.6	−0.0237
Moderate	16.7	7.3	0.2523	14.6	16.2	−0.0435
Moderate/severe	4.8	2.3	0.1143	3.2	4.1	−0.0380
Severe	18.5	4.0	0.3737	14.2	13.0	0.0313

ACEi, ACE inhibitors; AF, atrial fibrillation; ARB, angiotensin receptor blocker; ARNI, angiotensin receptor neprilysin inhibitor; ARVC, arrhythmogenic right ventricular cardiomyopathy; AS, aortic stenosis; AR, aortic regurgitation; BMI, body mass index; CI, Cardiac Index; DCM, dilated cardiomyopathy; HCM, hypertrophic cardiomyopathy; HR, heart rate; IHD, ischaemic heart disease; LAVi, left atrial maximal volume; LVEDP, left ventricular end-diastolic pressure; LVEF, left ventricular ejection fraction; MR, mitral regurgitation; MS, mitral stenosis; SBP, systolic blood pressure; PASP, pulmonary artery systolic pressure; PCWP, pulmonary capillary wedge pressure; PH, pulmonary hypertension; PM, pacemaker; RAP, right atrial pressure; RVEDP, right ventricular end-diastolic pressure; RVFAC, right ventricular fractional area change; RAVi right atrial maximal volume; TR, tricuspid regurgitation.

### Impact of atrial fibrillation on pulmonary capillary wedge pressure in the matched cohort

**Figure 1:**
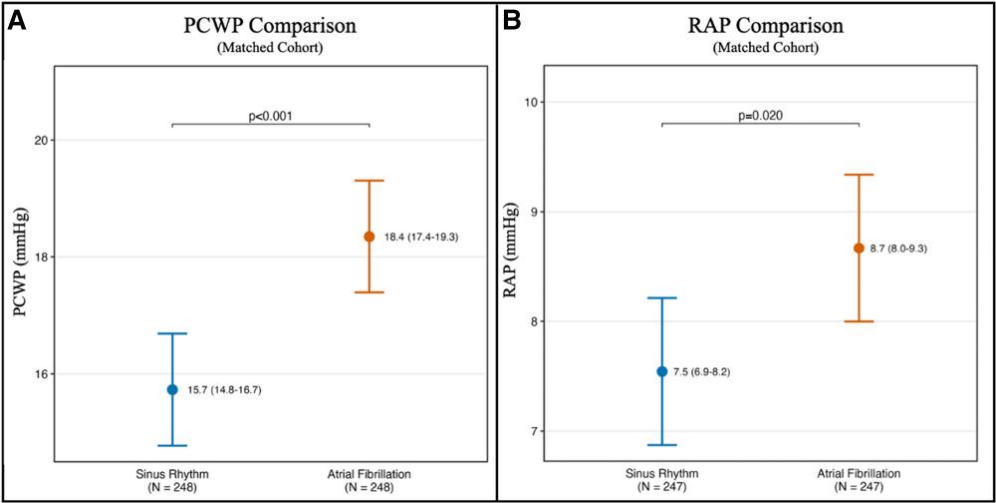
PCWP (*A*) and RAP (*B*) comparison in matched SR and AF groups

In the PCWP-matched cohort (*N* = 496), linear regression analysis demonstrated a significant association between AF and higher PCWP. The PCWP was 15.7 mmHg (95% CI: 14.8–16.7) for the SR group and 18.4 mmHg (95% CI: 17.4–19.3) for the AF group (*Figure 1A*, *Table 4*). Therefore, AF increased PCWP EMM by 2.6 mmHg (95% CI: 1.0–4.2; *P* < .001) compared to SR. Sensitivity analysis indicated that an unmeasured confounder would need a risk ratio of at least 10.86 with both AF and PCWP to nullify the observed estimate (E-value: 10.86). Furthermore, subgroup analysis confirmed that this association was consistent, although the magnitude was significantly increased by elevated SBP, older age, higher LVEF, and the presence of MS (Supplementary data online, *Figs*. *S5* and *S6*).

**Table 4:** Estimated effect of atrial fibrillation on pulmonary capillary wedge pressure

Effect of AF on PCWP (linear regression)				
	*Mean*	*SE*	*P-value*	*E-value*
**(Intercept)**	15.7	0.5	<.001	
**Atrial fibrillation**	2.6	0.7	<.001	10.86

Effect of AF on PCWP (EMM)				
	*Mean*	*SE*	*CI (95%)*	*P-value*
**Sinus rhythm**	15.7	0.5	14.8–16.7	<.001
**Atrial fibrillation**	18.4	0.5	17.4–19.3	

AF, atrial fibrillation; CI, confidence intervals; EMM, estimate marginal mean; PCWP, pulmonary capillary wedge pressure; and SE, standard error.

### Impact of atrial fibrillation on right atrial pressure in the matched cohort

In the RAP-matched cohort (*N* = 494), linear regression analysis demonstrated a significant association between AF and higher RAP. The RAP was 7.5 mmHg (95% CI: 6.9–8.2) for the SR group and 8.7 mmHg (95% CI: 8.0–9.3) for the AF group (*Figure 1B*, *Table 5*). Therefore, AF increased RAP EMM by 1.1 mmHg (95% CI: 0.2–2.1; *P* = .020) compared to SR. Sensitivity analysis indicated that an unmeasured confounder would need a risk ratio of at least 2.78 with both AF and RAP to nullify the observed estimate (E-value: 2.78). Furthermore, the effect of AF on RAP was consistent across confounders, with no evidence of significant effect modification (Supplementary  *Figs. S5* and *S6*).

**Table 5:** Estimated effect of atrial fibrillation on right atrial pressure

Effect of AF on RAP (linear regression)				
	*Mean*	*SE*	*P-value*	*E-value*
**(Intercept)**	7.5	0.4	<.001	
**Atrial fibrillation**	1.1	0.5	.020	2.78

Effect of AF on RAP (EMM)				
	*Mean*	*SE*	*CI (95%)*	*P-value*
**Sinus rhythm**	7.5	0.3	6.8–8.2	.020
**Atrial fibrillation**	8.7	0.3	8.0–9.3	

AF, atrial fibrillation; CI, confidence intervals; EMM, estimate marginal mean; PCWP, pulmonary capillary wedge pressure; and SE, standard error.

### Influence of atrial fibrillation history on atrial pressures

#### Analysis within the atrial fibrillation group

**Figure 2:**
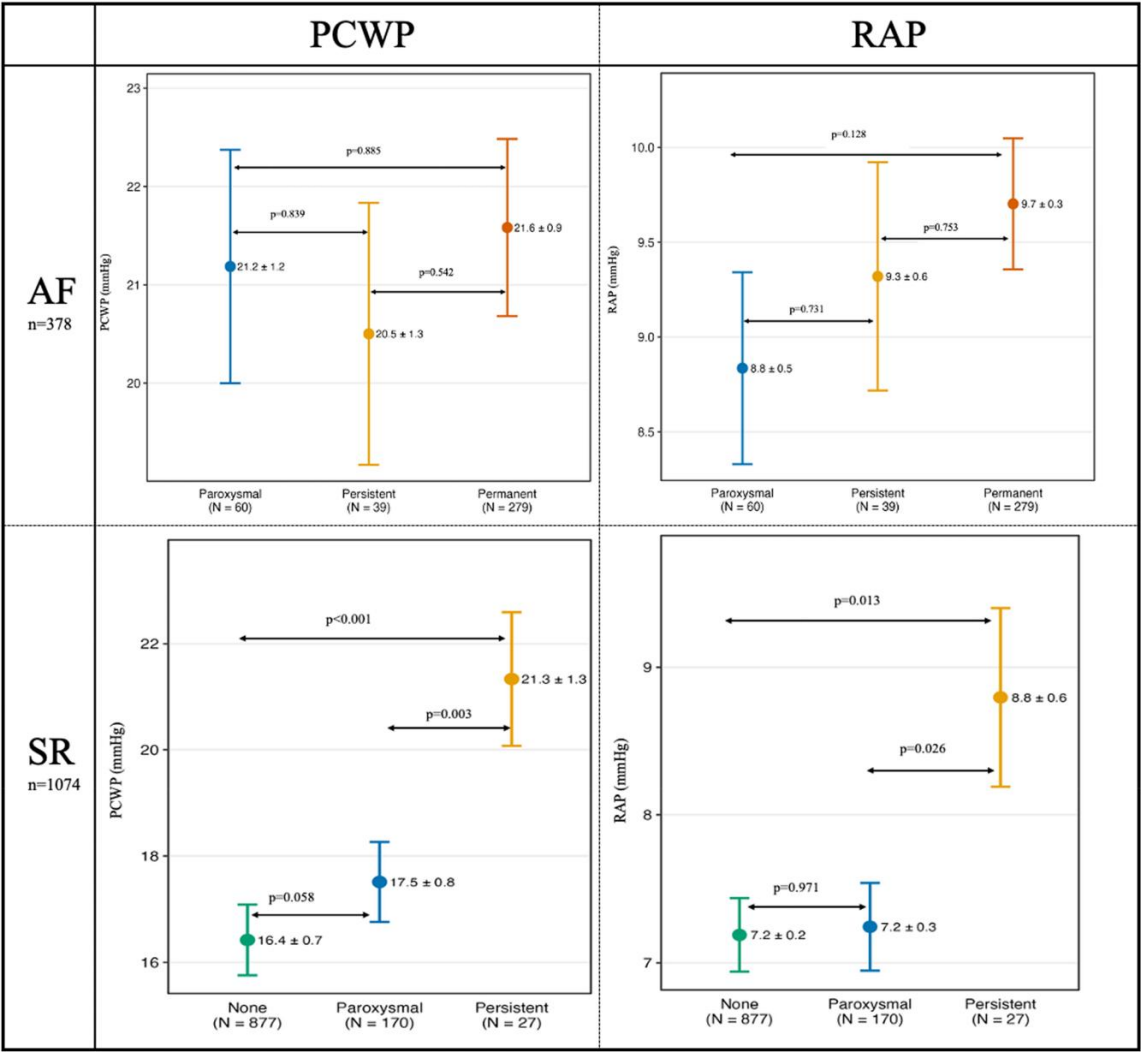
PCWP and RAP based on AF history in the AF group (*n* = 378) and SR group (*n* = 1074). See also Supplementary data online, *Tables S1–S6*. PCWP values accounting for age, BMI, cardiac disease, PM, haemoglobin, creatinine, albumin, beta-blockers, ACEiARB, furosemide, SBP, HR, CI, LVEDP, LVEF, LAVI, MR grade, and MS grade. RAP values accounting for age, BMI, cardiac disease, PM, haemoglobin, creatinine, albumin, beta-blockers, ACEiARB, furosemide, PASP, HR, CI, RVEDP, FAC; RAVI, TR grade

Among patients presenting in AF during RHC (*N* = 378 from the overall cohort), multiple linear regression adjusting for covariates explored the influence of AF temporal pattern (paroxysmal, persistent, permanent) on atrial pressures. No statistically significant differences were observed for PCWP (Paroxysmal: 21.2 ± 1.2 mmHg; Persistent: 20.5 ± 1.3 mmHg; and Permanent: 21.6 ± 0.9 mmHg) or RAP (Paroxysmal: 8.8 ± 0.5 mmHg; Persistent: 9.3 ± 0.6 mmHg; and Permanent: 9.7 ± 0.3 mmHg) among the different AF patterns (*Figure 2* and Supplementary data online, *Tables S1–S3*).

#### Analysis within the sinus rhythm group

Among patients presenting in SR during RHC (*N* = 1074 from the overall cohort), multiple linear regression adjusting for covariates examined the influence of AF temporal pattern (none, paroxysmal, persistent) on atrial pressures. Compared to patients with no history of AF (*N* = 877), those with a history of paroxysmal AF (*N* = 170) had comparable PCWP (17.5 ± 0.8 vs 16.4 ± 0.7 mmHg; *P* = .058) and RAP (7.2 ± 0.3 vs 7.2 ± 0.2 mmHg; *P* = .971). On the other side, patients with a history of persistent AF (*N* = 27) exhibited significantly higher PCWP and RAP in comparison to patients with a history of paroxysmal AF and no AF (PCWP: 21.3 ± 1.3 vs 17.5 ± 0.8 mmHg, *P* = .003, and vs 16.4 ± 0.7 mmHg, *P* < .001) (RAP: 8.8 ± 0.6 vs 7.2 ± 0.3 mmHg; *P* = .013 and vs 7.2 ± 0.2 mmHg; *P* = .013) (*Figure 2* and Supplementary data online, *Tables S4–S6*).

### Influence of atrial fibrillation on pulmonary capillary wedge pressure/right atrial pressure relationship

After adjusting the atrial pressures with the same list of confounders used for the PS analysis, the strength of the PCWP/RAP residuals relationship was not affected by AF, despite AF inducing a complete upward shift of the PCWP/RAP relationship in comparison to SR, resulting in a higher than expected PCWP for any given RAP when compared to SR (Supplementary data online, *Fig. S7*).

## Discussion

In this large cohort of patients with various chronic heart diseases, we found that AF increased PCWP by 2.6 mmHg and RAP by 1.1 mmHg after controlling for key clinical, echocardiographic, and haemodynamic confounders, highlighting the independent haemodynamic burden of AF disconnected from concomitant cardiac pathologies and comorbidities.

The observed independent effect of AF on PCWP confirms and expands on previous findings. A recent observational study demonstrated that PCWP was higher than LVEDP in AF patients. In contrast, it was the opposite in SR patients.^18^ However, these study findings were limited to a sole association between AF and the PCWP–LVEDP relationship, without controlling for any potential confounding factors. In fact, the SR and AF groups differed significantly in multiple covariates, including demographics, haemodynamics, cardiac diagnoses, and medications. Another retrospective study found AF, rheumatic valve diseases, and a larger left atrium as independent predictors for PCWP exceeding LVEDP.^19^ However, this study considered only a few confounders, examined AF's influence on PCWP–LVEDP in a dichotomous manner, and the data collected included variables measured from 6 months before to 6 months after RHC, which increases the risk that clinical changes might have occurred between measurements and RHC exams. The novelty of our study was to rigorously isolate and quantify the haemodynamic impact of AF on both atrial pressures using a dedicated dual-model propensity score matching approach, which enabled control over a wide range of related confounders. Therefore, while prior works have documented the existence of PCWP–LVEDP discordance in AF, our data provide a solid quantification of the direct haemodynamic effect of the arrhythmia itself, as AF independently raises PCWP by a mean of 2.6 mmHg compared to SR.

Canine models have shown that induced AF elevates both left and right atrial pressures,^20^ although these findings have not been firmly replicated in humans. In one study, AF was simulated by pacing simultaneously from the proximal coronary sinus and the His bundle region, attempting to artificially reproduce and isolate the components of AF's haemodynamic impact. It was suggested that loss of AV synchrony, irregular ventricular rate, and tachycardia might contribute to increased LAP.^21^ However, these findings were extrapolated from an artificial scenario caused by a pacing manoeuvre and only examined the immediate haemodynamic impact, which limits how directly they can be applied to real-world practice. Furthermore, in another study by the same authors, AF induction after rapid atrial pacing did not significantly increase LAP from SR.^22^

Our study provides robust evidence supporting AF's direct contribution to elevated atrial pressures independently of potential confounders, such as concomitant comorbidities and chronic cardiac pathologies, which are highly prevalent in AF. Despite the observational nature of the study, the high E-value (10.86) for the PCWP indicates robustness of the finding against potential unmeasured confounders. The strength of our study lies in analysing a large ‘real-world’ population with established chronic cardiac conditions, and our findings could offer a practical quantitative metric of AF haemodynamic impact to consider in everyday clinical practice. For example, the intrinsic pressure elevation solely due to AF provides a direct haemodynamic explanation for the higher natriuretic peptide levels frequently observed in AF patients.^23–25^

We also demonstrated that AF independently increases RAP, despite results showing a smaller effect (∼1.1 mmHg) and lower robustness (*E*-value 2.78) of the findings to unmeasured confounders compared to PCWP. Interestingly, because of the main focus of AF with PCWP, the impact of AF on RAP has rarely been reported before and has only been limited to animal studies,^20^ to artificial AF induction in EP lab environments,^26,27^ or not controlled for potential cofounders in observational studies.^28^ However, we believe that the haemodynamic impact of AF on RAP and PCWP is comparable since the smaller absolute increase in RAP compared to PCWP should be interpreted in light of their relative changes. The average PCWP in the AF group was 17% higher than in the SR group (18.4 vs 15.7 mmHg), and the average RAP was 16% higher (8.7 vs 7.5 mmHg), indicating a similar relative increase in pressure in both atria during AF.

An additional finding of our study was the influence of AF temporal patterns on atrial pressures.

The mechanisms of acute atrial pressure increase in AF are likely multifactorial, involving the loss of coordinated atrial systole, impaired ventricular filling, irregular ventricular response, and increased heart rate.^21^ In contrast, chronic remodelling contributes to the development of atrial cardiomyopathy, where electrical and mechanical dysfunction impairs atrial compliance, ultimately perpetuating AF persistence.^20,29^ This concept, recently defined in a clinical consensus, provides a key mechanistic framework for our findings.^30^ Interestingly, among patients presenting in AF, the temporal pattern (paroxysmal, persistent, permanent) did not influence atrial pressures after multivariable adjustment. This might suggest that the acute haemodynamic state during AF overrides differences related to the temporal pattern, or it could also reflect limitations in classifying AF temporal patterns. Conversely, among patients in SR at the time of RHC, we found a gradient of progressive increases in PCWP and RAP, from patients who had never had AF to those with a history of paroxysmal AF and to those with a history of persistent AF, despite this last finding should be interpreted with caution due to the small subgroup of persistent AF in SR at the time of exam (*n* = 27). This ‘dose–response’ relationship suggests that longer or more sustained exposure to AF causes lasting adverse atrial structural remodelling, which affects atrial pressures even during subsequent SR periods.^31,32^ This aligns with imaging studies demonstrating persistent atrial dysfunction in patients with a history of AF who are later in SR.^33^

### Clinical perspectives

The independent elevation of PCWP by approximately 2.6 mmHg and RAP by about 1.1 mmHg associated with AF, while numerically modest, may be crucial for accurately interpreting haemodynamic data, especially in patients with borderline values for diagnosing HF with preserved EF in the presence of AF. For example, a patient with a PCWP value of 14 mmHg during SR will fulfil the haemodynamic cut-off for HF during AF due to a PCWP increase over 15 mmHg.^3^ Furthermore, pulmonary hypertension patients with a similar PCWP value of 14 mmHg might be classified from pre-capillary to post-capillary during AF due to a PCWP increase over 15 mmHg.1 Our findings provide a quantifiable value (2.6 mmHg LAP and 1.1 mmHg RAP) that clinicians should consider when interpreting borderline atrial pressures in HF patients the presence of AF. Furthermore, the AF added haemodynamic burden could contribute to increased symptom severity, reduced exercise capacity, and worse prognosis in HF patients.^34,35^ Notably, even a slight chronic increase, with an amplitude similar to our findings, in intracardiac pressures is linked to a higher mortality risk in HF patients.^36^ Furthermore, the increased haemodynamic burden of the arrhythmia partially explains the higher natriuretic peptide levels observed during AF compared to SR in HF patients.^37,38^ Ultimately, these findings provide further pathophysiological rationale for considering more aggressive rhythm control strategies, aiming to alleviate this haemodynamic burden, reduce AF progression from paroxysmal to persistent/permanent, and ultimately improve symptoms and prognosis in patients with AF, as suggested by recent trials.^39,40^

### Limitations

First, as a retrospective, single-center study conducted at a tertiary referral hospital, potential selection and referral biases may exist, which could limit the generalizability of the findings and might provide limited insight into different cardiac diseases. Second, because of the observational design, we could identify a plausible causal relationship but could not definitively establish causality. Third, although PS matching and multivariable adjustments were applied, residual confounding from unmeasured or imperfectly measured variables cannot be entirely ruled out. Fourth, the classification of AF patterns was based on clinical history documented in medical records, which may not perfectly reflect the true arrhythmia burden or duration. Fifth, all subgroup analyses performed on the matched databases and those examining the impact of AF history on atrial pressures should be considered exploratory and hypothesis-generating, as these analyses may be limited by small sample sizes in certain subgroups (for example, the small sample size in the persistent AF subgroup within the SR patients). Sixth, mean RAP and PCWP values were measured during RHC; therefore, the results cannot be directly extrapolated to end-diastolic atrial pressures, as these measurements are not directly interchangeable. Seventh, echocardiographic evaluation of the atria was limited to volumes; future studies incorporating functional parameters, such as atrial strain, might offer further mechanistic insight into the observed pressure changes. Finally, the cross-sectional nature of data collection prevents analysis of temporal changes or the direct haemodynamic effects of AF onset or termination.

## Conclusions

In this large cohort of patients with various chronic heart diseases, AF independently increased both right and left atrial pressures. Furthermore, among patients in SR, a history of prior persistent AF was associated with higher atrial pressures. These findings highlight the intrinsic haemodynamic contribution of AF to atrial pressure elevation.

## Supplementary Material

xvaf032_Supplementary_Data

## Declarations

### Data Availability

No data were generated or analysed for this manuscript.

### Pre-registered Clinical Trial Number

None supplied.
