# Supplementary material for: Atrial fibrillation increases left and right atrial pressures in patients with chronic heart diseases
Source: ESC Heart Fail. 2026 Jan 8;13(1):xvaf032. doi: 10.1093/eschf/xvaf032 (PMC13108304; doi:10.1093/eschf/xvaf032)
Supplement: xvaf032_Supplementary_Data [file xvaf032_supplementary_data.docx]

**SUPPLEMENTARY MATERIAL**

**TABLES**

**Table S1.** Multivariate linear analysis for PCWP prediction in the AF subgroup

| **AF Subgroup, PCWP multivariate analysis** | | | | | |
| --- | --- | --- | --- | --- | --- |
|  | | **Estimate** | **SE** | **t value** | **p** |
| **Intercept** | | 4.619 | 5.724 | 807 | 0.420 |
| **AF History** | ***Persistent AF*** | -0.684 | 1.211 | -565 | 0.572 |
|  | ***Permanent AF*** | 0.396 | 0.839 | 472 | 0.637 |
| **Age (Years)** | | 0.062 | 0.034 | 1.865 | 0.063 |
| **BMI (kg/m²)** | | 0.079 | 0.068 | 1.167 | 0.244 |
| **Cardiac diagnosis** | ***DCM*** | -1.556 | 1.128 | -1.380 | 0.169 |
|  | ***PH*** | 0.880 | 1.830 | 481 | 0.630 |
|  | ***AS*** | 0.675 | 1.110 | 608 | 0.543 |
|  | ***AR*** | -0.365 | 3.018 | -121 | 0.903 |
|  | ***MS*** | 3.643 | 1.918 | 1.900 | 0.058 |
|  | ***MR*** | -1.475 | 1.371 | -1.076 | 0.282 |
|  | ***TR*** | -0.743 | 1.394 | -533 | 0.595 |
|  | ***HCM*** | 0.993 | 1.955 | 508 | 0.612 |
|  | ***ARVC*** | -1.293 | 3.611 | -358 | 0.720 |
|  | ***Others*** | 0.330 | 1.942 | 170 | 0.865 |
| **PM** | | 0.090 | 0.832 | 109 | 0.914 |
| **Hemoglobin (g/dL)** | | -0.109 | 0.189 | -575 | 0.566 |
| **Creatinine (µmol/L)** | | 0.007 | 0.008 | 880 | 0.379 |
| **Albumin (g/L)** | | -0.010 | 0.067 | -142 | 0.887 |
| **Beta-Blocker** | | -0.209 | 0.670 | -299 | 0.765 |
| **ACEi/ARB/ARNI** | | 0.960 | 0.667 | 1.438 | 0.151 |
| **Furosemide (mg/die)** | | 0.006 | 0.003 | 2.278 | 0.023 |
| **SBP (mmHg)** | | 0.038 | 0.014 | 2.630 | 0.008 |
| **HR (bpm)** | | 0.036 | 0.020 | 1.768 | 0.077 |
| **CI (L/min/m²)** | | -1.065 | 0.517 | -2.061 | 0.040 |
| **LVEDP (mmHg)** | | 0.432 | 0.047 | 9.156 | < 0.001 |
| **LVEF (%)** | | -0.078 | 0.0270 | -2.882 | 0.004 |
| **LAVol Max (mL/m²)** | | 0.019 | 0.009 | 2.181 | 0.030 |
| **MR** | ***Mild*** | 3.245 | 0.991 | 3.274 | 0.001 |
|  | ***Mild/Moderate*** | 0.721 | 0.829 | 871 | 0.384 |
|  | ***Moderate*** | 0.318 | 0.870 | 366 | 0.715 |
|  | ***Moderate/Severe*** | 0.714 | 0.875 | 815 | 0.415 |
|  | ***Severe*** | 0.395 | 0.856 | 461 | 0.645 |
| **MS** | ***Mild*** | 4.173 | 1.472 | 2.835 | 0.005 |
|  | ***Moderate*** | -0.453 | 0.987 | -459 | 0.647 |
|  | ***Severe*** | 1.267 | 1.039 | 1.219 | 0.223 |

**Table S2.** Multivariate linear analysis for RAP prediction in the AF subgroup

| **AF Subgroup, RAP multivariate analysis** | | | | | |
| --- | --- | --- | --- | --- | --- |
|  | | **Estimate** | **SE** | **t value** | **p** |
| **Intercept** | | 0.917 | 2.876 | 319 | 0.750 |
| **AF History** | ***Persistent AF*** | 0.484 | 0.642 | 754 | 0.451 |
|  | ***Permanent AF*** | 0.867 | 0.445 | 1.946 | 0.053 |
| **Age (Years)** | | -0.016 | 0.017 | -933 | 0.351 |
| **BMI (kg/m²)** | | 0.068 | 0.036 | 1.896 | 0.059 |
| **Cardiac diagnosis** | ***IHD*** | 1.380 | 0.606 | 2.279 | 0.023 |
|  | ***DCM*** | 1.211 | 0.960 | 1.266 | 0.206 |
|  | ***PH*** | 1.334 | 0.569 | 2.346 | 0.019 |
|  | ***AS*** | 1.961 | 1.577 | 1.244 | 0.214 |
|  | ***AR*** | 0.359 | 0.682 | 527 | 0.598 |
|  | ***MS*** | 1.254 | 0.632 | 1.984 | 0.048 |
|  | ***MR*** | 2.705 | 0.793 | 3.410 | <0.001 |
|  | ***TR*** | 0.915 | 0.991 | 923 | 0.357 |
|  | ***HCM*** | 3.553 | 1.965 | 1.808 | 0.071 |
|  | ***ARVC*** | 0.077 | 1.017 | 76 | 0.940 |
| **PM** | | 0.592 | 0.423 | 1.400 | 0.163 |
| **Hemoglobin (g/dL)** | | -0.322 | 0.100 | -3.214 | 0.001 |
| **Creatinine (µmol/L)** | | 0.002 | 0.004 | 527 | 0.598 |
| **Albumin (g/L)** | | 0.060 | 0.034 | 1.756 | 0.080 |
| **Beta-Blocker** | | 0.281 | 0.372 | 756 | 0.450 |
| **ACEi/ARB/ARNI** | | 0.283 | 0.345 | 820 | 0.413 |
| **Furosemide (mg/die)** | | -0.001 | 0.001 | -686 | 0.493 |
| **HR (bpm)** | | 0.001 | 0.011 | 130 | 0.897 |
| **RVEDP (mmHg)** | | 0.606 | 0.040 | 15.351 | < 0.001 |
| **PASP (mmHg)** | | 0.065 | 0.013 | 4.969 | <0.001 |
| **Cardiac Index (L/min/m²)** | | 0.055 | 0.268 | 205 | 0.838 |
| **RAVolMax (mL/m²)** | | -0.005 | 0.006 | -809 | 0.419 |
| **RVFAC (%)** | | -0.041 | 0.018 | -2.305 | 0.022 |
| **TR** | ***Mild*** | 2.152 | 0.528 | 4.084 | <0.001 |
|  | ***Mild/Moderate*** | 1.367 | 0.4224 | 3.236 | 0.001 |
|  | ***Moderate*** | -0.464 | 0.485 | -958 | 0.339 |
|  | ***Moderate/Severe*** | 0.027 | 0.508 | 54 | 0.957 |
|  | ***Severe*** | -0.325 | 0.431 | -755 | 0.450 |

**Table S3.** AF subgroup. PCWP and RAP values for paroxysmal, persistent and permanent AF.

| **AF GROUP** | | | | | | | | |
| --- | --- | --- | --- | --- | --- | --- | --- | --- |
|  | **AF history** | **Mean** | **SE** | **CI [95%]** | **AF History Comparison** | **Mean Comparison** | **SE** | **p** |
| **PCWP** | **Paroxysmal** | 21.2 | 1.190 | 18.9-23.5 | **Paroxysmal - Persistent** | 0.685 | 1.210 | 0.839 |
|  | **Persistent** | 20.5 | 1.330 | 17.9-23.1 | **Paroxysmal - Permanent** | -0.396 | 0.839 | 0.885 |
|  | **Permanent** | 21.6 | 0.901 | 19.8-23.4 | **Persistent - Permanent** | -1.081 | 1.020 | 0.542 |
| **RAP** | **Paroxysmal** | 8.84 | 0.506 | 7.84-9.83 | **Paroxysmal - Persistent** | -0.484 | 0.642 | 0.731 |
|  | **Persistent** | 9.32 | 0.602 | 8.14-10.50 | **Paroxysmal - Permanent** | -0.867 | 0.445 | 0.128 |
|  | **Permanent** | 9.70 | 0.346 | 9.02-10.38 | **Persistent - Permanent** | -0.382 | 0.534 | 0.753 |

**Table S4.** Multivariate linear analysis for PCWP prediction in the SR subgroup

| **SR Subgroup, PCWP multivariate analysis** | | | | | |
| --- | --- | --- | --- | --- | --- |
|  | | **Estimate** | **SE** | **t value** | **p** |
| **Intercept** | | 5.125 | 2.924 | 1.753 | 0.080 |
| **AF History** | ***Paroxysmal AF*** | 1.090 | 0.477 | 2.286 | 0.022 |
|  | ***Persistent AF*** | 4.911 | 1.094 | 4.492 | <0.001 |
| **Age (Years)** | | -0.012 | 0.016 | -770 | 0.442 |
| **BMI (kg/m²)** | | 0.223 | 0.037 | 6.107 | <0.001 |
| **Cardiac diagnosis** | ***DCM*** | -1.827 | 0.669 | -2.731 | 0.006 |
|  | ***PH*** | 1.306 | 0.802 | 1.627 | 0.104 |
|  | ***AS*** | -1.122 | 0.628 | -1.788 | 0.074 |
|  | ***AR*** | -0.059 | 0.917 | -64 | 0.949 |
|  | ***MS*** | 1.043 | 1.591 | 656 | 0.512 |
|  | ***MR*** | -2.310 | 0.980 | -2.357 | 0.019 |
|  | ***TR*** | -0.061 | 1.565 | -39 | 0.969 |
|  | ***HCM*** | 0.959 | 1.332 | 720 | 0.471 |
|  | ***ARVC*** | -1.779 | 1.763 | -1.009 | 0.313 |
|  | ***Others*** | -1.008 | 1.301 | -775 | 0.439 |
| **PM** | | 0.052 | 0.686 | 75 | 0.940 |
| **Hemoglobin (g/dL)** | | -0.004 | 0.040 | -91 | 0.927 |
| **Creatinine (µmol/L)** | | 0.009 | 0.002 | 372 | 0.710 |
| **Albumin (g/L)** | | -0.024 | 0.037 | -633 | 0.527 |
| **Beta-Blocker** | | 0.714 | 0.366 | 1.954 | 0.051 |
| **ACEi/ARB/ARNI** | | 0.019 | 0.373 | 50 | 0.960 |
| **Furosemide (mg/die)** | | 0.007 | 0.002 | 2.944 | 0.003 |
| **SBP (mmHg)** | | 0.015 | 0.007 | 2.106 | 0.035 |
| **HR (bpm)** | | 0.036 | 0.013 | 2.848 | 0.005 |
| **Cardiac Index (L/min/m²)** | | -0.676 | 0.242 | -2.793 | 0.005 |
| **LVEDP (mmHg)** | | 0.381 | 0.024 | 15.623 | < 0.001 |
| **LVEF (%)** | | -0.087 | 0.019 | -4.669 | <0.0013 |
| **LAVolMax (mL/m²)** | | 0.042 | 0.011 | 3.677 | <0.001 |
| **MR** | ***Mild*** | 2.645 | 0.742 | 3.566 | <0.001 |
|  | ***Mild/Moderate*** | -0.323 | 0.562 | -575 | 0.565 |
|  | ***Moderate*** | 1.654 | 0.590 | 2.806 | 0.005 |
|  | ***Moderate/Severe*** | 1.147 | 0.629 | 1.823 | 0.068 |
|  | ***Severe*** | -1.287 | 0.649 | -1.983 | 0.048 |
| **MS** | ***Mild*** | 4.868 | 1.166 | 4.174 | <0.001 |
|  | ***Moderate*** | 0.597 | 0.883 | 676 | 0.499 |
|  | ***Severe*** | 0.097 | 0.769 | 126 | 0.900 |

**Table S5.** Multivariate linear analysis for RAP prediction in the SR subgroup

| **SR Subgroup, RAP multivariate analysis** | | | | | |
| --- | --- | --- | --- | --- | --- |
|  | | **Estimate** | **SE** | **t value** | **p** |
| **Intercept** | | 2.651 | 1.420 | 1.867 | 0.062 |
| **AF History** | ***Paroxysmal AF*** | 0.055 | 0.239 | 230 | 0.818 |
|  | ***Persistent AF*** | 1.607 | 0.569 | 2.826 | 0.005 |
| **Age (Years)** | | -0.010 | 0.008 | -1.334 | 0.182 |
| **BMI (kg/m²)** | | 0.079 | 0.019 | 4.159 | <0.001 |
| **Cardiac diagnosis** | ***IHD*** | 0.391 | 0.332 | 1.179 | 0.238 |
|  | ***DCM*** | -1.516 | 0.379 | -3.999 | <0.001 |
|  | ***PH*** | 0.1211 | 0.290 | 417 | 0.676 |
|  | ***AS*** | -0.089 | 0.449 | -198 | 0.843 |
|  | ***AR*** | 0.262 | 0.622 | 421 | 0.674 |
|  | ***MS*** | -0.342 | 0.366 | -936 | 0.349 |
|  | ***MR*** | 0.349 | 0.889 | 392 | 0.695 |
|  | ***TR*** | 2.260 | 0.654 | 3.457 | <0.001 |
|  | ***HCM*** | 2.097 | 0.919 | 2.283 | 0.023 |
|  | ***ARVC*** | 0.523 | 0.652 | 803 | 0.422 |
| **PM** | | 0.113 | 0.343 | 330 | 0.741 |
| **Hemoglobin (g/dL)** | | -0.005 | 0.020 | -241 | 0.809 |
| **Creatinine (µmol/L)** | | 0.002 | 0.001 | 1.287 | 0.198 |
| **Albumin (g/L)** | | -0.026 | 0.019 | -1.385 | 0.166 |
| **Beta-Blocker** | | 0.016 | 0.1836 | 86 | 0.932 |
| **ACEi/ARB/ARNI** | | 0.193 | 0.187 | 1.037 | 0.300 |
| **Furosemide (mg/die)** | | 0.002 | 0.001 | 1.417 | 0.157 |
| **HR (bpm)** | | 0.001 | 0.006 | 157 | 0.876 |
| **RVEDP (mmHg)** | | 0.3716 | 0.019 | 19.344 | < 0.001 |
| **PASP (mmHg)** | | 0.052 | 0.007 | 7.159 | <0.001 |
| **Cardiac Index (L/min/m²)** | | -0.122 | 0.123 | -989 | 0.322 |
| **RAVolMax (mL/m²)** | | -0.006 | 0.007 | -831 | 0.406 |
| **RVFAC (%)** | | -0.023 | 0.011 | -2.147 | 0.032 |
| **TR** | ***Mild*** | 2.811 | 0.431 | 6.522 | <0.001 |
|  | ***Mild/Moderate*** | 1.154 | 0.347 | 3.331 | <0.001 |
|  | ***Moderate*** | -0.491 | 0.373 | -1.317 | 0.188 |
|  | ***Moderate/Severe*** | -0.381 | 0.376 | -1.012 | 0.312 |
|  | ***Severe*** | -0.138 | 0.344 | -402 | 0.688 |

**Table S6** SR subgroup. PCWP and RAP values for none, paroxysmal and persistent AF.

| **SR SUBGROUP** | | | | | | | | |
| --- | --- | --- | --- | --- | --- | --- | --- | --- |
|  | **AF history** | **Mean** | **SE** | **CI [95%]** | **AF History Comparison** | **Mean Comparison** | **SE** | **p** |
| **PCWP** | **None** | 16.4 | 0.664 | 15.1-17.7 | **None - Paroxysmal** | -1.09 | 0.477 | 0.058 |
|  | **Paroxysmal** | 17.5 | 0.752 | 16.0-19.0 | **None - Persistent** | -4.91 | 1.090 | <0.001 |
|  | **Persistent** | 21.3 | 1.260 | 18.9-23.8 | **Paroxysmal - Persistent** | -3.82 | 1.160 | 0.003 |
| **RAP** | **None** | 7.19 | 0.248 | 6.70-7.68 | **None - Paroxysmal** | -0.55 | 0.239 | 0.971 |
|  | **Paroxysmal** | 7.24 | 0.296 | 6.66-7.82 | **None - Persistent** | -1.607 | 0.568 | 0.013 |
|  | **Persistent** | 8.80 | 0.606 | 7.61-9.99 | **Paroxysmal - Persistent** | -1.552 | 0.596 | 0.026 |

**Supplementary Figure 1:** PCWP Analysis. PS distribution before and after PS matching

**
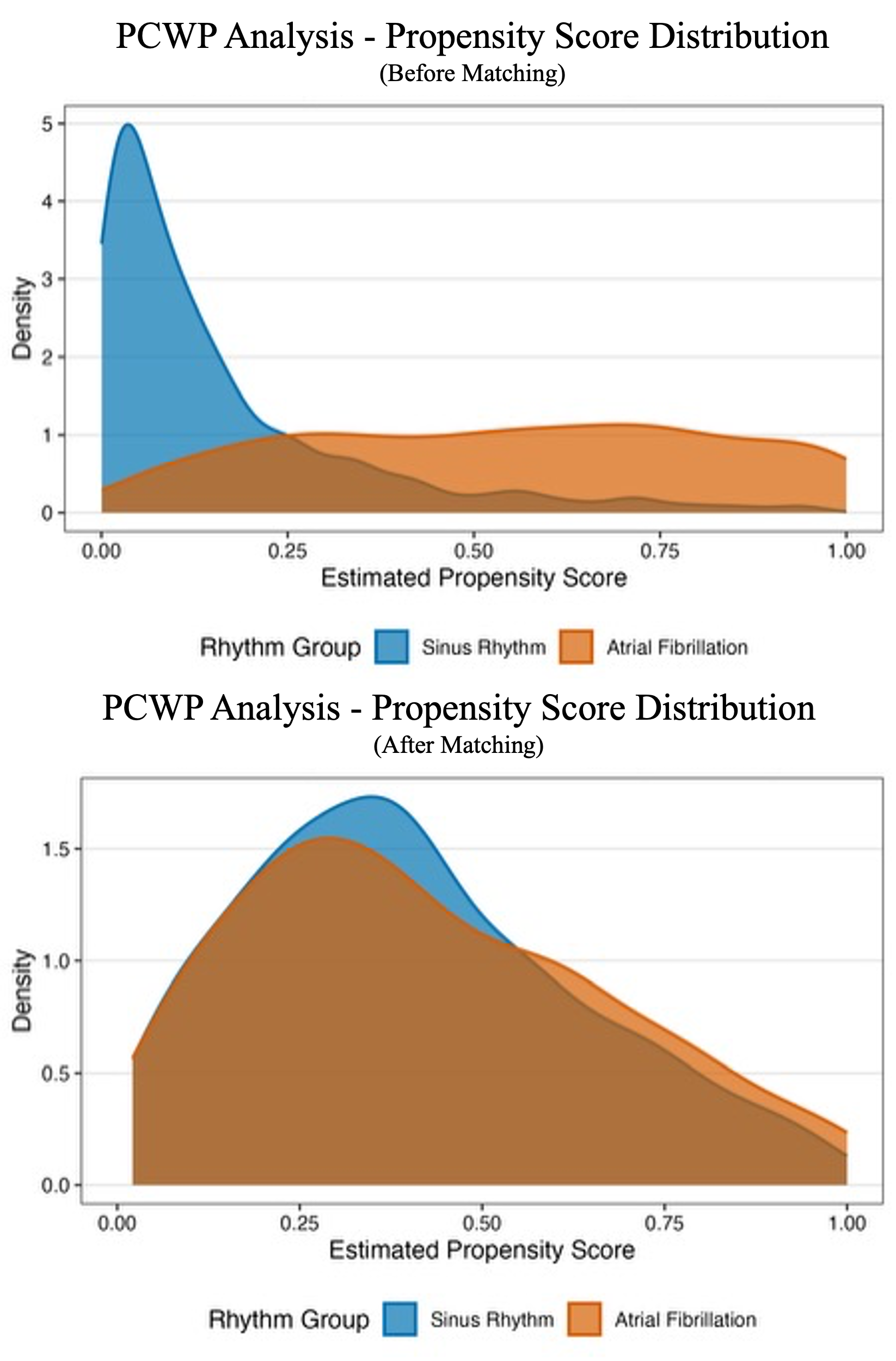
**

**Supplementary Figure 2:** PCWP Analysis. Covariate balance before and after PS matching.

**
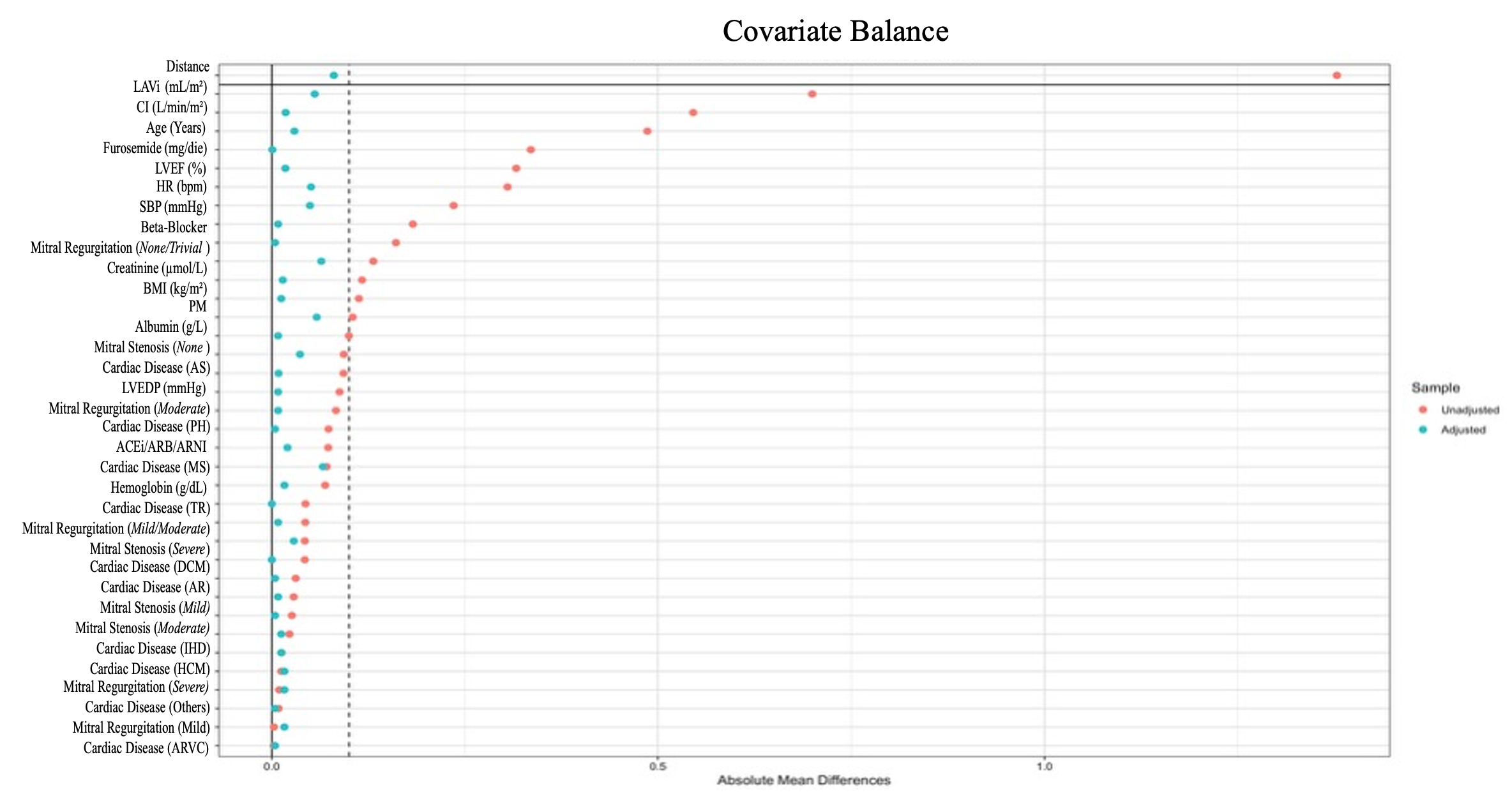
**

**Supplementary Figure 3:** RAP Analysis. PS distribution before and after PS matching.

**
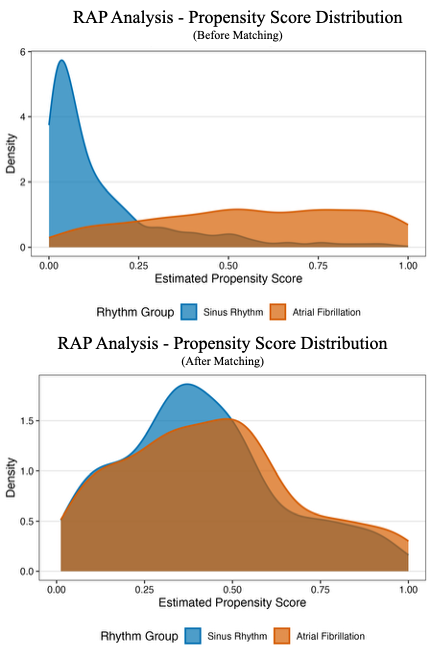
**

**Supplementary Figure 4:** RAP Analysis. Covariate balance before and after PS matching.

**
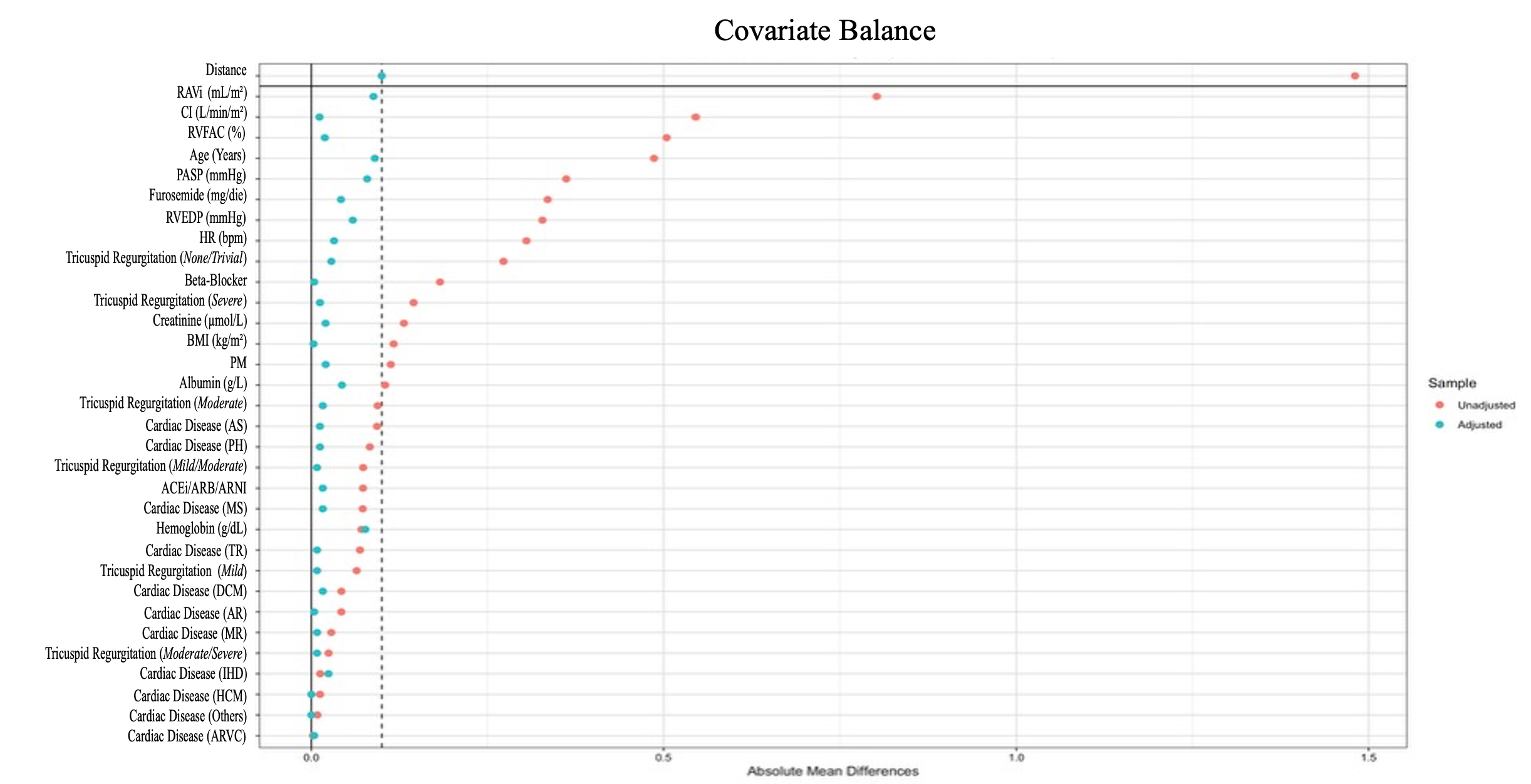
**

**Supplementary Figure 5:** PCWP and RAP subgroup analysis in the matched cohorts

**
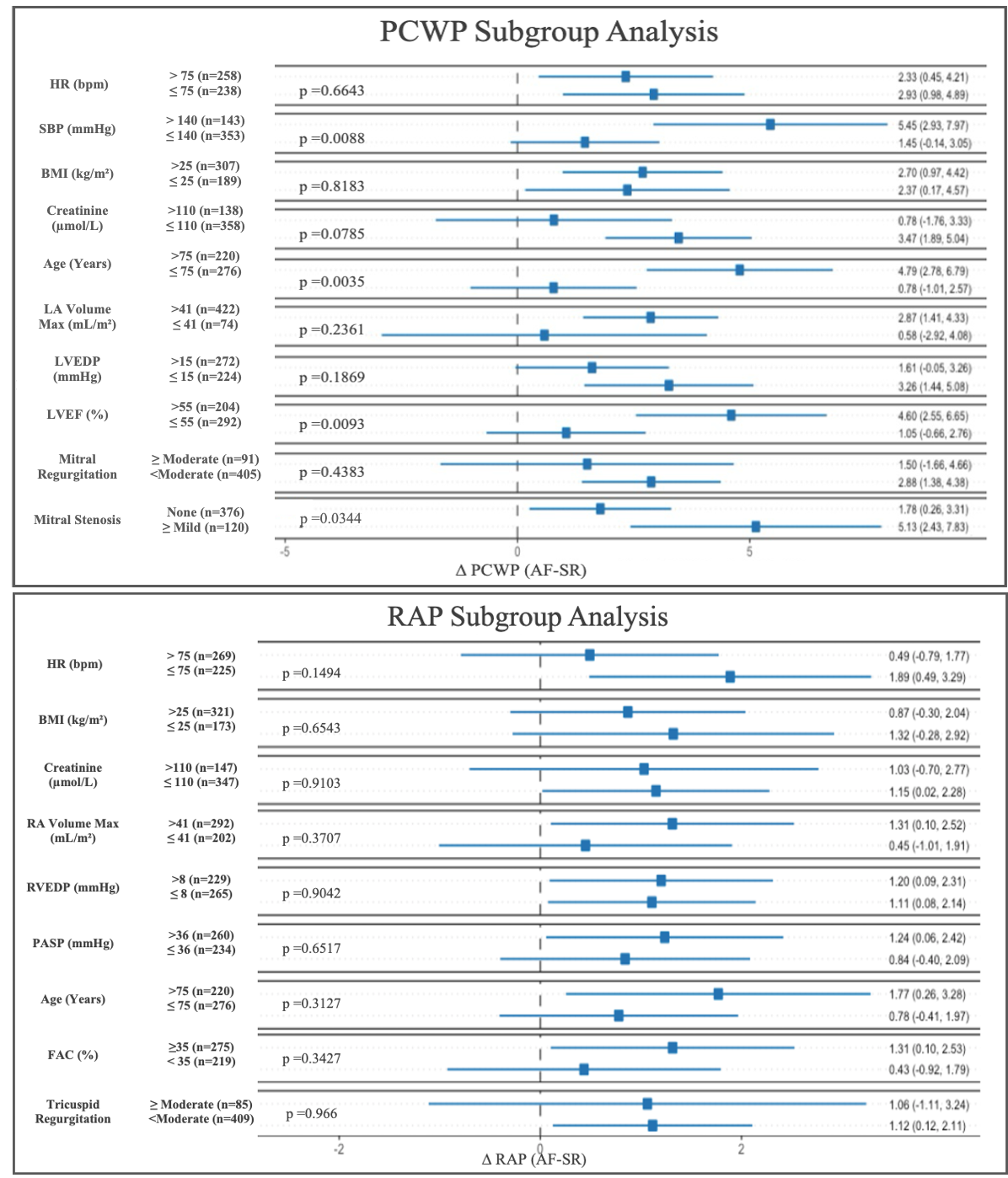
**

**Supplementary Figure 6:** PCWP and RAP stratified by main cardiac diagnosis in the matched cohort


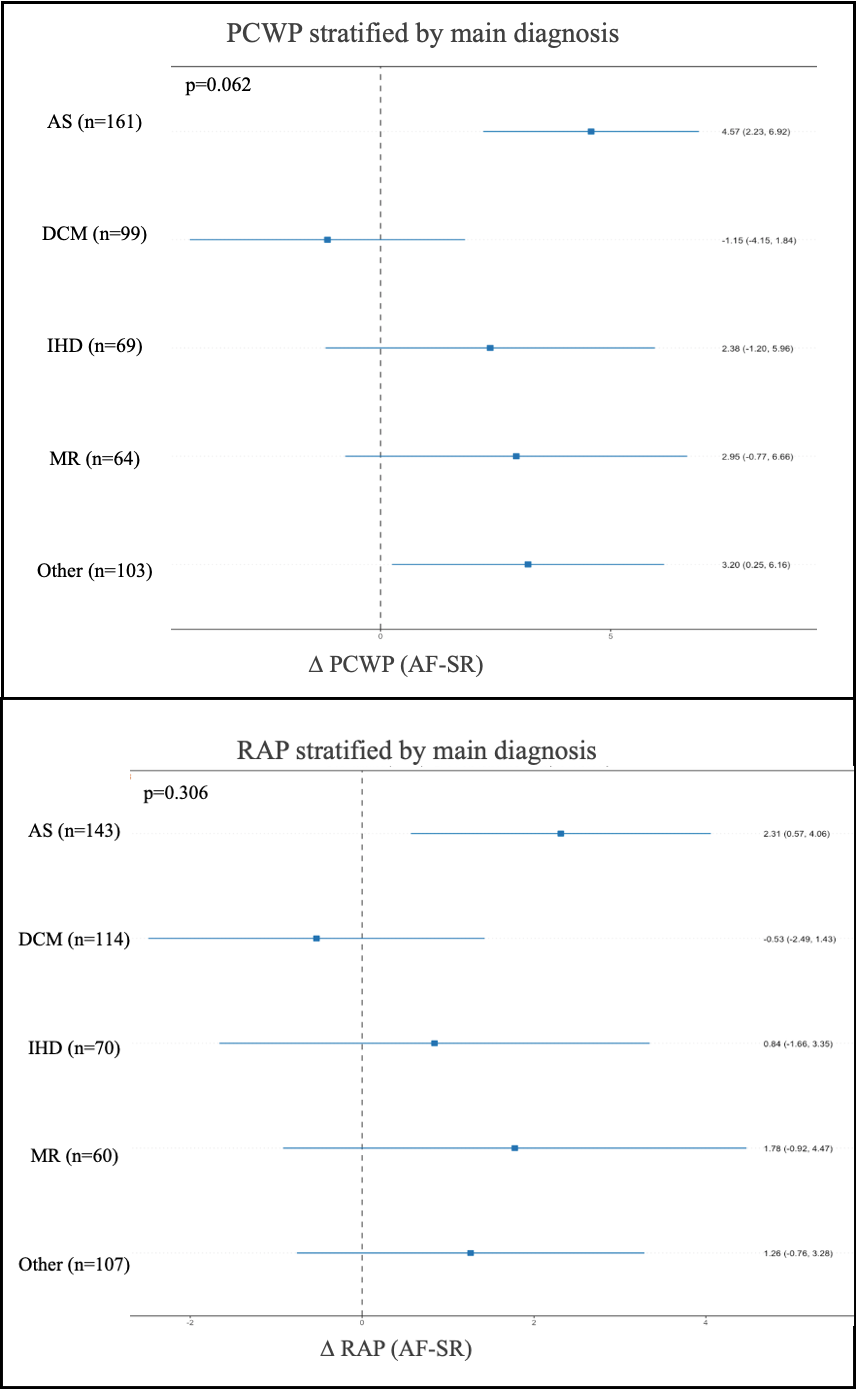


**Supplementary Figure 7:** Influence of AF on PCWP/RAP relationship. Unadjusted analysis (A) and adjusted analysis (B) of PCWP and RAP residuals after adjustment for their corresponding confounders.

**
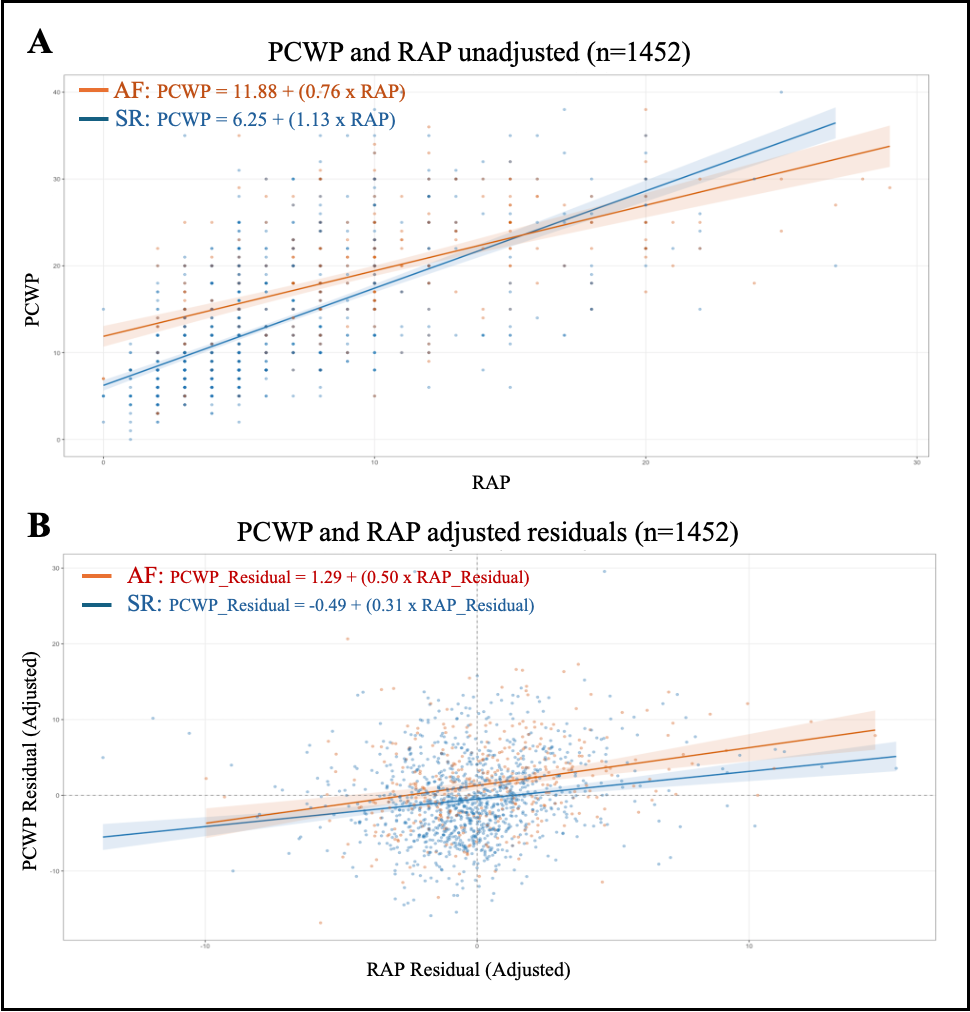
**

PCWP residuals obtained after adjusting for: Age, BMI, Cardiac disease, PM, Hemoglobin, Creatinine, Albumin, Betablockers, ACEiARB, Furosemide, SBP, HR, CI, LVEDP, LVEF, LAVI, MR grade, MS grade.

RAP residuals obtained after adjusting: Age, BMI, Cardiac disease, PM, Hemoglobin, Creatinine, Albumin, Betablockers, ACEiARB, Furosemide, PASP, HR, CI, RVEDP, FAC; RAVI, TR grade.
